# Characterization of Lignin Compounds at the Molecular Level: Mass Spectrometry Analysis and Raw Data Processing

**DOI:** 10.3390/molecules26010178

**Published:** 2021-01-01

**Authors:** Ruochun Zhang, Yulin Qi, Chao Ma, Jinfeng Ge, Qiaozhuan Hu, Fu-Jun Yue, Si-Liang Li, Dietrich A. Volmer

**Affiliations:** 1 Institute of Surface-Earth System Science, School of Earth System Science, Tianjin University, Tianjin 300072, China; zhangruochun@tju.edu.cn (R.Z.); machao2019@tju.edu.cn (C.M.); 2019231001@tju.edu.cn (J.G.); hqz_2020@tju.edu.cn (Q.H.); fujun_yue@tju.edu.cn (F.-J.Y.); siliang.li@tju.edu.cn (S.-L.L.); 2 Tianjin Key Laboratory of Earth Critical Zone Science and Sustainable Development in Bohai Rim, Tianjin University, Tianjin 300072, China; 3 Department of Chemistry, Humboldt-Universität zu Berlin, 12489 Berlin, Germany; dietrich.volmer@hu-berlin.de

**Keywords:** lignin, mass spectrometry, ionization, data processing, structural information

## Abstract

Lignin is the second most abundant natural biopolymer, which is a potential alternative to conventional fossil fuels. It is also a promising material for the recovery of valuable chemicals such as aromatic compounds as well as an important biomarker for terrestrial organic matter. Lignin is currently produced in large quantities as a by-product of chemical pulping and cellulosic ethanol processes. Consequently, analytical methods are required to assess the content of valuable chemicals contained in these complex lignin wastes. This review is devoted to the application of mass spectrometry, including data analysis strategies, for the elemental and structural elucidation of lignin products. We describe and critically evaluate how these methods have contributed to progress and trends in the utilization of lignin in chemical synthesis, materials, energy, and geochemistry.

## 1. Introduction

Lignin is the second most abundant natural polymer after cellulose and a sustainable biomass [1]. It consists of approximately 20% of grasses and straws, 30% of softwoods, and 25% of poplar, which plays crucial roles in the plants’ nutrition transport and shape maintenance [2]. Lignin polymers are composed of phenylpropanoids, primarily coniferyl, sinapyl and coumaryl alcohols, which are connected by different linkages, e.g., the most abundant β-*O*-4′ linkage [3]. Lignin structures are complex, greatly depending on the lignin sources and the methods for separation [4]. 

The high natural abundance of lignin makes it one of the most promising renewable materials [5]. Biomass is regarded as a promising renewable source of energy, which could substitute traditional fossil fuels [6]. Lignocellulosic biomass is mainly composed of cellulose, hemicellulose, and lignin, and the three components exhibit different properties and pyrolytic behaviors [7]. The relative abundances of the three components play a crucial role in the biomass quality after pyrolysis. For example, it has been reported that parent biomass with lower lignin content normally produces high quality bio-oil [8]. Therefore, separation and characterization of the biomass at the molecular level before and after pyrolysis are required to optimize the transformation processes and to extract the proper compounds [8]. Breaking down lignin while preserving its aromatic nature (which yields the most useful content) has the potential to provide a value stream of material that is currently exclusively obtained from petroleum sources [9]. 

In addition to energy-related uses, lignin is available at a low cost as a by-product of the pulp and paper industry as well as the cellulosic ethanol producing processes, which makes it an attractive alternative to polyacrylonitrile for the production of carbon fibers and the production of anodes for lithium-ion batteries [10]. Moreover, lignin can also be used for the production of aromatic fine chemicals and oligomers [11]. Applications and technologies obtained from lignin-derived products (e.g., concrete additives, industrial binders, and biopolymers for ceramics) indicate that conversion of lignin into smaller molecules can be a profitable and sustainable industry [12]. Chemical characterization of the product mixtures is essential for optimizing the lignin conversion processes, but it remains very challenging, because these mixtures are complex and contain isomers with a wide variety of functional groups. 

Conventional analytical methods, such as gas chromatography-mass spectrometry (GC-MS), nuclear magnetic resonance (NMR), and Fourier transform infrared spectroscopy (FTIR) have been applied to the characterization of lignin and lignin-derived compounds [13,14,15]. However, NMR and FTIR are capable mostly only of the identification of functional groups. On the other hand, compounds with high boiling points and molecular weights are difficult to be analyzed by GC or GC-MS [16,17]. Therefore, a further understanding of lignin mixtures requires not only advanced techniques, but also the combination of multiple techniques. Comprehensive two-dimensional GC coupled to flame ionization detection (GC×GC-FID) or time-of-flight MS (GC×GC-TOF) show capability for the characterization of complex mixtures such as bio-oils. In addition, Fourier transform ion cyclotron resonance mass spectrometry (FT-ICR MS) is currently regarded as a powerful technique for characterizing the mixtures or heavy components of biomass, crude oil, and natural organic matters [18,19,20,21]. 

FT-ICR MS has also been applied for lignin analysis since the 1990s [22]. The technique offers high broadband mass resolution (>300,000) and mass accuracy (<1 ppm), which enables accurate elemental assignments of tens of thousands of compounds present in a complex sample such as lignin and bio-oils from lignocellulosic biomass [23,24]. Multiple ionization methods, such as electrospray ionization (ESI) [25] and atmospheric-pressure photoionization (APPI) [26], have been used with FT-ICR MS or other high-resolution MS (HRMS) techniques for the characterization of bio-oil, lignin, and lignin-derived compounds.

Finally, lignin is one of the most commonly employed molecular tracers for terrestrial organic matters in the marine environment [27]. Its structural composition has been extensively investigated to characterize the source and transformation of geological deposits [28,29,30]. However, it is challenging to assess the structure of lignin due to its large and insoluble features. Lignin is traditionally regarded as a very stable compound, which is insensitive to biological and chemical degradation. However, increasing evidence points to the fact that lignin is not as stable as usually assumed [31,32]. It has been demonstrated that lignin phenols exhibit various degradation processes due to weathering in the environment, which are affected by the polymer composition, size, crosslinking, and functional groups. For instance, angiosperm-derived syringyl phenols and non-woody-tissue-derived cinnamyl phenols are reported to decay faster than vanillyl phenols [33,34]. Considering the significance of lignin in industrial and geological application, a better understanding of lignin compounds at the molecular level is vital. The primary objective of this review is to provide comprehensive information on the MS analysis of lignin. Specifically, we summarized the literature regarding ionization techniques, tandem MS procedures, and data-processing methods for lignin analysis.

## 2. Role of Ionization Technique on Compounds Coverage

Atmospheric pressure ionization (API) techniques are most commonly used for the mass spectrometric study of lignin compounds. However, as the lignin components vary in size, heteroatom content, number of functional groups, etc., there is no universal ionization technique or MS instrument setting that will analyze each of these components with equal efficiency.

**Figure 1:**
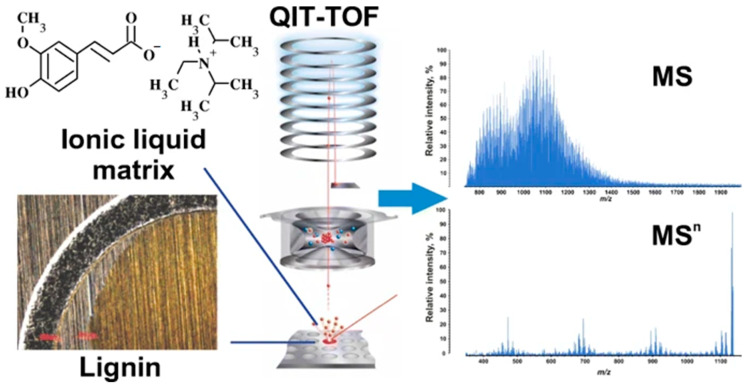
Ionic liquid matrices for matrix-assisted laser desorption/ionization (MALDI) mass spectrometry of lignin produced high-quality MS and MS*^n^* mass spectra. Reprinted from Kosyakov et al., with permission from Springer Publishing [40].

The mass spectra of a lignin sample measured by various ion sources often differ drastically in the spectral appearance, due to unknown methodological problems [35]. Above all, selection of the proper ionization conditions is the key factor for obtaining high-quality mass spectra of natural compounds. Compared to API, matrix-assisted laser desorption/ionization (MALDI) is not popular in lignin studies, mostly because of its low ionization efficiency using conventional matrices, which prevents the reproducible acquisition of high-quality mass spectra [36,37]. Nevertheless, MALDI is a pulsed ionization technique acquiring spectra within milliseconds; for this reason, MALDI can still be successfully applied as a shotgun method to quickly visualize the most abundant compounds in various lignin samples [38]. Kosyakov et al. compared the efficiencies of various crystalline matrices and their mixtures for MALDI analysis of lignin. The authors studied the effect of matrix-to-analyte ratio on the quality of the mass spectrum [39]. α-Cyano-4-hydroxycinnamic (CHCA), 2,5-dihydroxybenzoic acid (DHB), and 2,4,6-trihydroxyacetophenone (THAP) were found to be the best matrices used in 10–100 folds excess with respect to the lignin sample. The authors also proposed for the first time that the use of ionic liquids formed a homogeneous solution with lignin, which gave substantially better results (Figure 1). Subsequently, the same group tested 32 ionic liquids consisting of nitrogen-containing cations and anions of aromatic acids and found that the use of such matrices in combination with the MALDI quadrupole ion trap-TOF MS provided high-intensity mass spectra of lignin. Additionally, the utilization of MS^2^ and MS^3^ detected various precursor ions for the first time (Figure 1). In addition, it is important to point out that lignins are aromatic compounds, which can serve as light-absorbing chemical matrices themselves. Recently, Qi et al. compared common MALDI matrices as well as direct laser ionization (no MALDI matrix) for the analysis of alkali lignin and discovered that different MALDI matrices exhibited very unique selectivities for sulfur- and nitrogen-containing lignin species [38]. Therefore, all the above studies clearly demonstrate that the choice of MALDI matrix should be tailored to meet particular measurement purposes to cover the desired compound classes.

**Figure 2:**
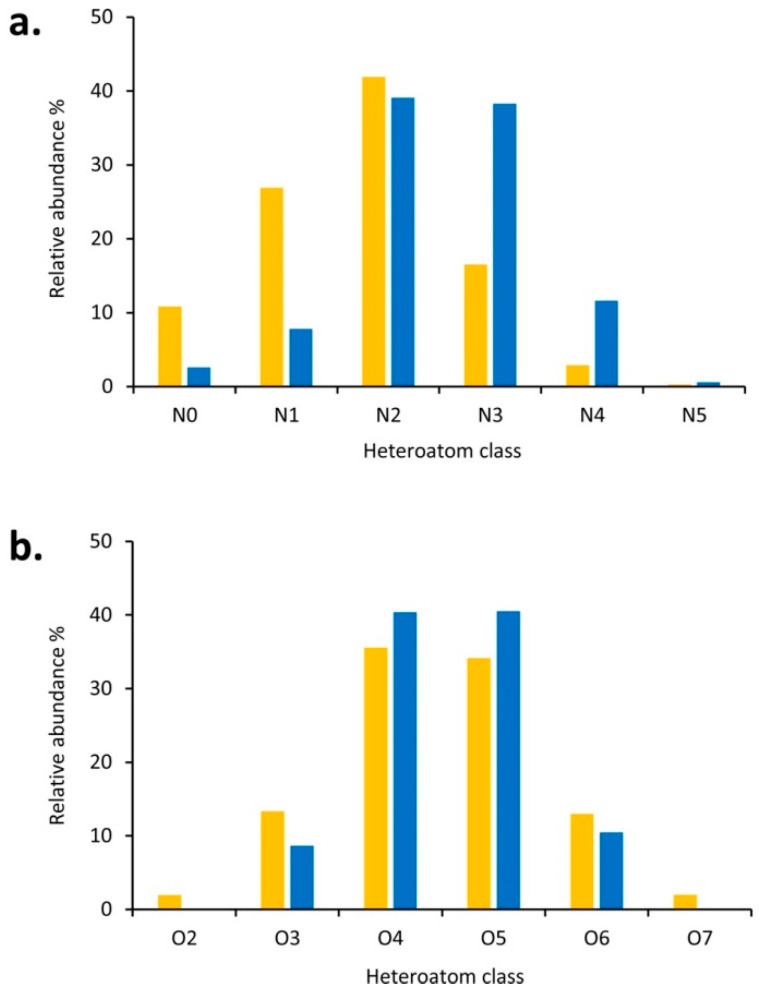
Relative distributions of the positive ions for different compound classes observed in the study of a pyrolysis bio-oil when ammonia (yellow) or ammonium acetate (blue) was added with respect to (**a**) the number of nitrogen atoms for CxHyN_0–5_O_2–7_ compounds and (**b**) the number of oxygen atoms for CxHyO_2–7_ species. Reprinted from Hertzog et al., with permission from the American Chemical Society [23].

For more detailed and comprehensive analyses, several API sources are usually implemented. Given the presence of a large abundance of hydroxyl, carboxyl, and phenolic groups, ESI under negative ionization conditions is the most widely applied ionization method for lignin [32]. Hertzog et al. explored the influence of the sample preparation and the choice of dopant on the measurement of a pyrolysis lignin-derived bio-oil by ESI in both positive and negative ion modes [23]. A significant number of nitrogen-containing species were detected, when ammonia, ammonium acetate, or formic acid was added (Figure 2). This study showed the importance of a well-controlled composition of the sample solution to ensure both sensitivity and repeatability for the ESI measurement.

**Figure 3:**
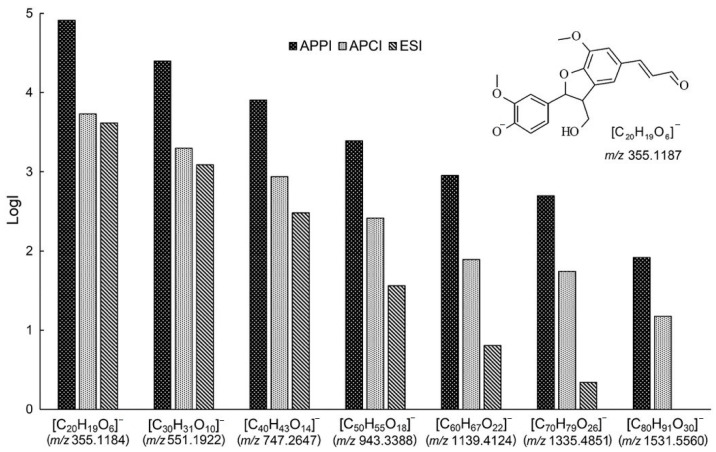
Comparison of the peak intensity (LogI) of lignin oligomers in the mass spectra obtained by electrospray ionization (ESI), atmospheric pressure chemical ionization (APCI) and atmospheric-pressure photoionization (APPI). Reprinted from Kosyakov et al. with permission from Wiley [26].

Alternatively, atmospheric pressure chemical ionization (APCI) is often considered as the main alternative to ESI in studies of lignin [41]. In APCI, the sample is evaporated first and ionized in the gas phase by means of a corona discharge. This method is able to analyze small, lesser polar, more volatile molecules than ESI, often with lower matrix effects. Unfortunately, biopolymers also undergo partial fragmentation under the harsher APCI conditions. Banoub et al. were the first to propose APPI for the study of wheat straw lignin [42]. APPI initiates the ionization process by photon irradiation using a Krypton lamp. Compounds appropriate for this technique include larger aromatic molecules, whereas gases and solvents are not ionized by APPI, which greatly minimizes background interference. Kosyakov et al. compared the efficiency and characteristics of the three API techniques ESI, APCI, and APPI in the negative ionization mode using commercial lignin standards [26]. The authors characterized APPI as the preferred means for studying lignin, due to its higher signal intensities (Figure 3) and a lower interference levels of contaminants. It is important to point out that the optimum sensitivity was achieved at high liquid flow rates of ca. 0.2–1 mL/min for both APCI and APPI, resulting in much higher sample consumption as compared to ESI.

More recently, Qi et al. systematically evaluated the performance of three API techniques using a commercial lignin standard [43]. The data clearly showed that the number of heteroatoms (e.g., N, S, and P) in the sample greatly increased the chemical diversity of lignin, and the number of observed components was significantly influenced by the ionization technique used. The authors recommended that comprehensive ionization methods should be applied to interpret a lignin sample to ensure broad characterization as well as to prevent erroneous interpretations.

## 3. Tandem Mass Spectrometry Procedures

The molecular structure is the foundation to explore and utilize high-value lignin compounds. Various degradation procedures and/or catalytic pathways are required to convert lignin into smaller molecules in order to increase its energy density [44,45]. Unfortunately, these transformations result in very complex mixtures, and the biggest challenge is to match specific structures or chemical functionalities to measured elemental formulae. 

For linear biopolymers such as proteins, primary structures can be uniquely defined by the one-dimensional sequences, whereas for lignin compounds, full structural characterization requires multi-dimensional determinations: linkages, functional groups, stereo-chemical configurations, and even structural isomers.

Currently, tandem mass spectrometry (MS*^n^*) is the only analytical method for the molecular-level analysis of complex mixtures without using time-consuming purification steps [46,47]. Until now, the analysis of lignin by MS has mostly focused on monomeric compounds based on pyrolysis-gas chromatography (Py-GC)/MS analysis [48,49]. However, this approach tends to produce simple phenols by disrupting the lignin structure and removing its chemical functionalities.

**Figure 4:**
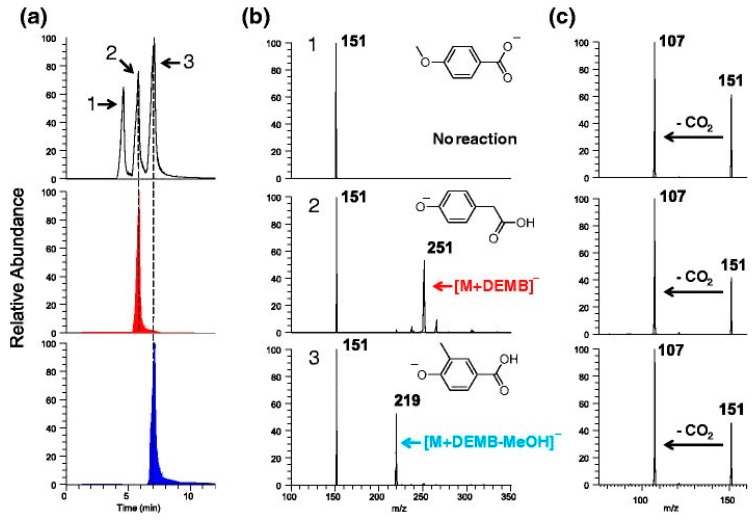
(**a**) (top) Total ion HPLC chromatogram measured for an equimolar mixture of 4-methoxybenzoic acid (1), 4-hydroxyphenylacetic acid (2), and 4-hydroxy-3-methylbenzoic acid (3). (**b**) MS^2^ spectra measured after the reaction of 4-methoxybenzoic acid (top), 4-hydroxyphenacetic acid (middle), and 4-hydroxy-3-methylbenzoic acid (bottom) with diethylmethoxyborane. (**c**) Collision-induced dissociation (CID) spectra measured for 4-methoxybenzoic acid (top), 4-hydroxyphenacetic acid (middle), and 4-hydroxy-3-methylbenzoic acid (bottom). Reprinted from Zhu et al. with permission from the Springer Publishing [56].

In recent years, several methods based on API in combination with high-performance liquid chromatography (HPLC) and MS*^n^* have been developed for the investigation of lignin samples from different environmental sources. Detailed elemental compositions, analyte classes, as well as in-depth structure information were proposed for some typical components, which permitted thorough molecular-level characterization of unknown mixtures derived from degraded lignin [42,50,51]. Banoub and co-workers provided an informative tutorial article, which summarized the application of MS*^n^* for lignin sequencing [52]. Many published MS methods were restricted to high-resolution data only for MS^2^ experiments in multiple-stage tandem MS experiments. However, it was shown that molecular weights and valuable structural information can be obtained for lignin-type molecules by subjecting them to multiple consecutive ion isolation and collision-induced dissociation (CID) cycles up to MS^7^ [53]. In CID, ions of interest are isolated and subjected to collisions, converting part of the collisional kinetic energy into ions’ internal energy and initializing fragmentation, which can provide information on the ion’s functional groups and structural units [54]. The fragment ions can be further isolated and subjected to more collisions to obtain increased structural information (MS*^n^*). CID enabled the identification of characteristic reaction pathways and the delineation of reasonable fragmentation mechanisms for deprotonated molecules containing various functional groups. Based on a comparison of fragmentation patterns to model compounds of lignin, Kenttamaa et al. utilized LC-MS*^n^* to provide structural information for components of a lignin degradation mixture (Figure 4); the method even permitted the differentiation of simple structural isomers such as eugenol and isoeugenol, 4-methoxybenzoic acid, 4-hydroxyphenylacetic acid, and 4-hydroxy-3-methylbenzoic acid (Figure 4) [51,55,56].

Subsequently, the CID fragmentation pathways of 34 small lignin model compounds representing the degradation products were explored experimentally and computationally by the same group. To further investigate the various fragmentation patterns, multistage MS*^n^* (*n* up to 6) was utilized to probe the product ions of the model compounds and their fragments; quantum–chemical calculations were employed to examine proposed reaction mechanisms. It was demonstrated that the resulting information can help to identify the presence of specific functionalities (e.g., carboxylic acid, aldehyde, ester, phenol groups) in a lignin sample [57]. In addition, the Kenttamaa group also summarized the CID patterns and key fragment ions of model compounds with β-*O*-4 and/or 5-5 linkages to facilitate the sequencing of unknown lignin oligomers, which revealed the number of specific linkages [58].

In the field of proteomics, LC-MS*^n^* produces protein fragmentation information, and computational algorithms are able to perform alignments with amino acids from known databases to predict the protein’s sequence with a degree of certainty. Unfortunately, no MS libraries exist for the lignin. Therefore, elucidating the MS*^n^* fragmentation mechanism of the major bonding types encountered in lignin-associated compounds would considerably benefit the lignin identification.

Structural databases for the lignin can also be obtained via artificial compounds synthesized in vitro by the polymerization of individual monomers. Kiyota et al. established a simple way to synthesize lignin, which mimics the natural polymerization process. Then, the resulting compounds can be analyzed via LC-MS*^n^* to determine their structures and to build a library of lignin oligomer data; the so-called “do-it-yourself” database was able to determine the overall composition and recalcitrance of biomass [59]. Morreel et al. also succeeded in annotating typical fragmentations for the β-aryl ether, benzodioxane, phenylcoumaran, and resinol groups, and they enabled the identification of aromatic units involved in each bonding structure [50,60]. Banoub et al. also utlized CID to study the structure the phenylcoumaran derivatives, which was unambiguously assigned as the protonated 4-carboxyl-coniferyl-(beta-5′)-(3-methoxylbenzene) unit, forming the cyclic ether dimer constituent unit of all phenylcoumaran derivatives.

**Figure 5:**
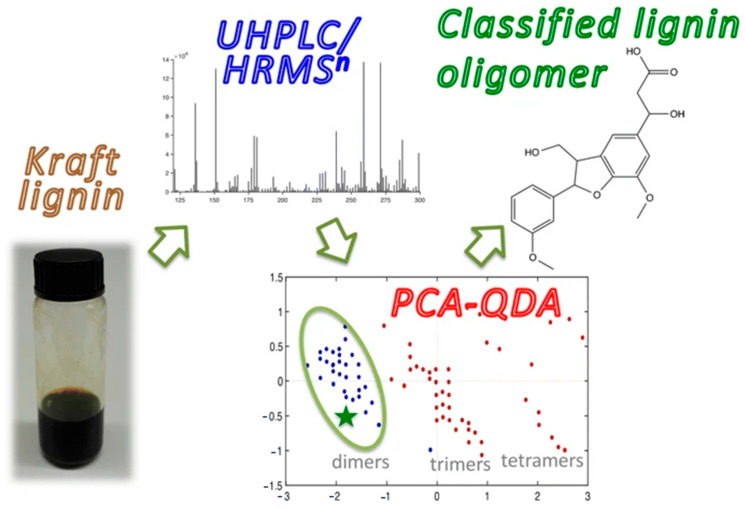
Workflow for a non-targeted LC-MS*^n^* strategy. Reprinted from Prothmann et al., with permission from the Springer Publishing [61].

More recently, Prothmann et al. presented a non-targeted LC-MS*^n^* strategy for the identification of lignin oligomers in Kraft lignin [61]. The identification confidence for oligomers was improved by introducing two pre-selection steps: Data-dependent neutral loss MS^3^ in combination with a principal component analysis-quadratic discriminant analysis (PCA-QDA) classification model for the oligomers (Figure 5). High-resolution data were acquired for all experimental stages, and structures of the identified compounds from the complex samples were deduced based on LC-MS*^n^* experiments, without the need for chemical standards. With the above method, 36 tentative oligomers were identified from 587 peaks in the Kraft lignin sample, consisting of lignin dimers, trimers, and tetramers.

## 4. Graphical and Statistical Methods for the HRMS Data

Considering the immense complexity of lignin mixtures, LC-MS*^n^* is the most straightforward way to distinguish the structural information of both native and degraded lignins. Unfortunately, due to cost and low throughput levels, most LC-MS and MS*^n^* protocols are not well suited for obtaining detailed data on tens of thousands of individual components with relative abundances that vary over several orders of magnitude. Consequently, data processing is required to simplify the datasets as well as to explore the hidden information from the complex mass spectra.

Nowadays, numerous graphical and statistical methods have been developed in the study of natural organic compound, to simplify the data acquired from HRMS instruments. For example, in the field of petroleomics, the Kendrick mass defect (KMD) permits the rescaling of the compounds’ mass-to-charge (*m/z*) ratio according to their homologous structural units, which provides an alignment of thousands of compounds with the same functional groups [62]. Both carbon number distribution and double-bond equivalent (DBE) plots have also been utilized to evaluate the structural features of crude oil acquired from different environmental sources. Additionally, the van Krevelen plot helps to sort the same classes of compounds in specific regions within the diagram and makes it straightforward to visualize possible links between individual molecules [63,64]. All of these approaches have been extended to the interpretation of polymers and lignins [65,66].

**Figure 6:**
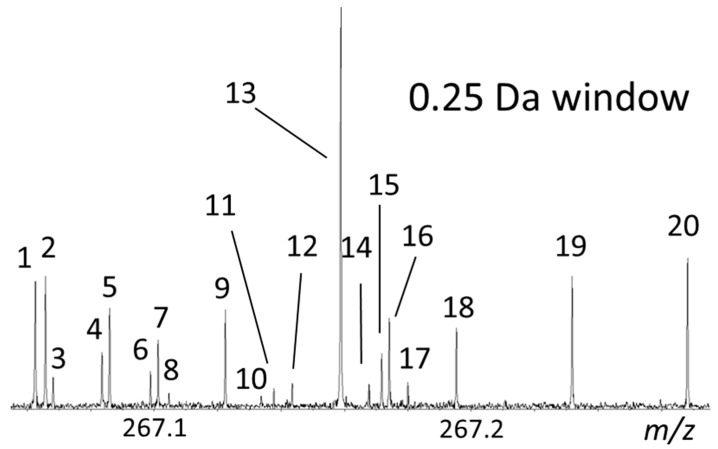
Mass-scale-expanded segments (0.25 u) of lignin broadband mass spectra. Reprinted from Qi et al. with permission from Elsevier [43].

For example, oxidative and reductive decomposition during the electrochemical degradation of lignin produces a large number of unknown products. From the mass spectral raw data, more than 5000 elemental compositions could be assigned in a single full-scan mass spectrum using high-resolution FT-ICR MS [3,67]. As an example, the complexities and peak densities in a region of only 0.25 *m*/*z* unit from a single full-scan mass spectrum of a lignin degradation mixture was expanded, exhibiting a total of 20 assigned features in this small segment (Figure 6) [43]. Even though each peak in the spectrum represented a chemically distinct compound, their molecular structures still remained unknown.

**Figure 7:**
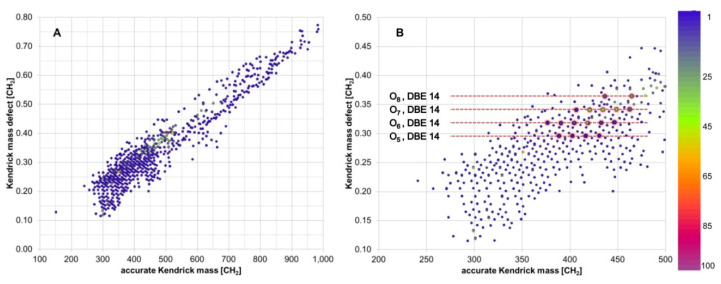
Kendrick mass defect (KMD) plots of a degraded lignin sample for (**A**) all measured *m*/*z* features; (**B**) restricted to monomeric/dimeric/trimeric content. Color scale represents relative intensity. Reprinted from Dier et al., with permission from the American Chemical Society [68].

Considering the immense complexity of these mass spectral datasets, simplification and classification methods are clearly required. In recent years, Volmer’s group modified the visualization methods from petroleomics research, to better adapt them to lignin samples [68,69]. At a first glance of the data, the classic KMD can be utilized to interrogate the mass spectra and to visualize hidden information (Figure 7). A linked *m/z* series with identical KMD appear as horizontal lines, normalized to 14 u (the CH_2_ unit), and therefore, the differences of mass defect along a vertical line are able to provide information on the oxygen content of the sample; that is, higher KMD values show higher oxygen contents. The degradation products were mostly observed in the *m/z* range 200–500, showing the formation of monomeric to trimeric lignin units, and a longer alkylation series of related substance classes within a narrow KMD band illustrate controlled breakdown reactions to common substance classes, e.g., oxidized resinols (O_7_, DBE = 11), oxidized β-*O*-4 linked products (O_6_, DBE = 10), and 5–5 linked compounds (O_6_, DBE = 9; O_6_, DBE = 11).

**Figure 8:**
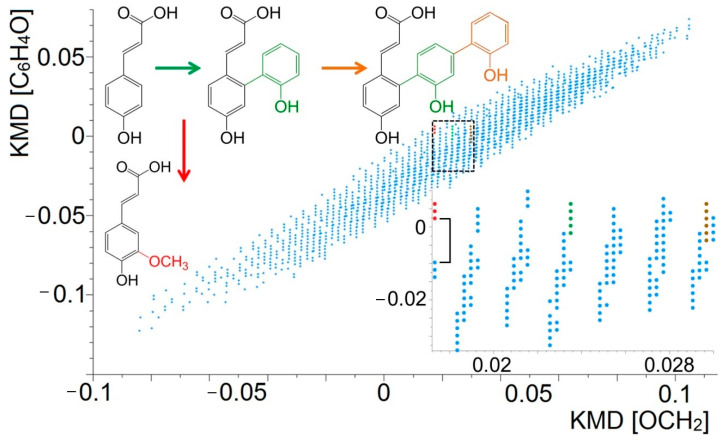
Two-dimensional (2D) mass defect matrix plot for a lignin sample after decomposition. Blue data points represent features in the KMD plot and correspond to degradation products from the sample. The squared area is enlarged (inset) and proposed core structures of the three compound species (red, green, brown) are highlighted in the expanded plot. Reprinted from Qi et al., with permission from Springer Publishing [69].

As mentioned above, thousands of elemental compositions could be assigned in a single full-scan mass spectrum of lignin. Due to isobaric inferences (Figure 6), it was impossible to isolate most of the low abundant ion signals for the subsequent MS*^n^* analyses, even though they could be clearly detected. To explore the bulk of the features contained in the datasets, Qi et al. expanded the concept of KMD plots to so-called “two-dimensional (2D) mass defect plots”, which allowed detailed interpretation of the observed signals together with their structural information [69]. Utilizing homologous functional groups and structural units of lignin as complementary *m/z* scaling factors provided further possibilities for simplifying the complex mass spectra. By using structure-specific metrics, the 2D matrix plots provided systematic line-ups of the different lignin linkages. Candidate *m/z* values and chemical structures were deduced from the genealogical links between products and their formation mechanisms, rather than unsystematically assigned chemical formulae in the conventional elemental composition analysis. Starting from the low *m/z* region (mainly consisting of monomers), the backbone structures of lignin could be easily identified via MS*^n^*. Based on the structural core, the oligomer structures with higher molecular weight originating from the same linkages were quickly filtered according to the mass defect base applied in the matrix plots. Then, the advantage of the plot is that structures of higher molecular weights but lower abundances in the samples (which are intense enough for MS*^n^* analysis) could be predicted on the basis of this information. For example, Figure 8 illustrates a small region that was further expanded from the 2D matrix plot. In the expanded view, three series of data points are highlighted with red, green, and brown colors. The measured *m/z* value for the first (red) data point was at *m/z* 163.0389. The structure of this precursor ion was confirmed to be coumaryl acid by the MS*^n^* experiment. The two following related red points on the vertical *y*-axis indicated that up to two methoxy (OCH_3_) groups can be assigned to the compound. Similarly, on the *x*-axis, the green and brown data points were both aligned with the coumaryl alcohol series horizontally, which indicated that they shared the same core structure. Further MS*^n^* experiments revealed that the structures of these compounds were linked by phenyl additions to the coumaryl acid core. Apart from the horizontal and vertical axes in the 2D plot, the slopes of the slanted lines also have diagnostic potential. Reactions that involve a loss or gain of a specific functional group can be identified from these trend lines, as in theory, each reaction pathway has its own trend line with characteristic slopes and intercepts.

**Figure 9:**
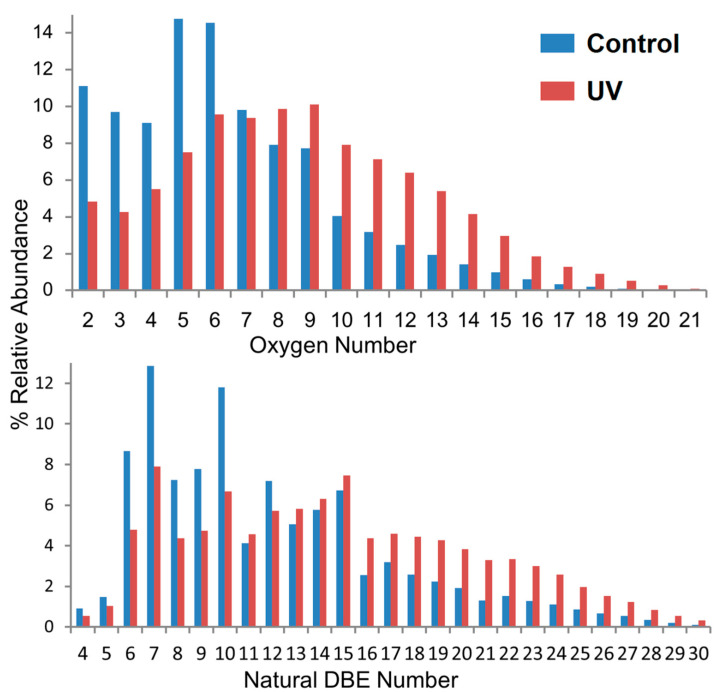
Relative abundances of oxygen-containing compounds (top) and double-bond equivalent (DBE) distribution (bottom) of lignins in control and oxidized samples. The relative abundance is the individual compound mass spectral peak abundance divided by the summed magnitudes of all peaks in the spectrum. Reprinted from Qi et al., with permission from Springer Publishing [32].

The utilization of economically viable biomass from lignin requires detailed knowledge on the lignin precursor materials, including the chemical transformations from weathering processes. Graphical methods can also be applied to study the effects of these degradation processes of lignin samples [70], including van Krevelen diagrams, bar charts, KMD, DBE, and carbon number plots. Figure 9 illustrates that generally, oxidation occurred extensively for different classes of lignin compounds, especially for those with a higher number of oxygen atoms [32]. DBE abundance plots additionally showed that small molecules with a single benzene ring were particularly sensitive to light, whereas multi-aromatic rings protected the compounds from photodegradation. Surprisingly, relative abundances of compounds with DBE < 10 remained almost unchanged, which may enable these compounds to be used as markers for the original lignin species.

**Figure 10:**
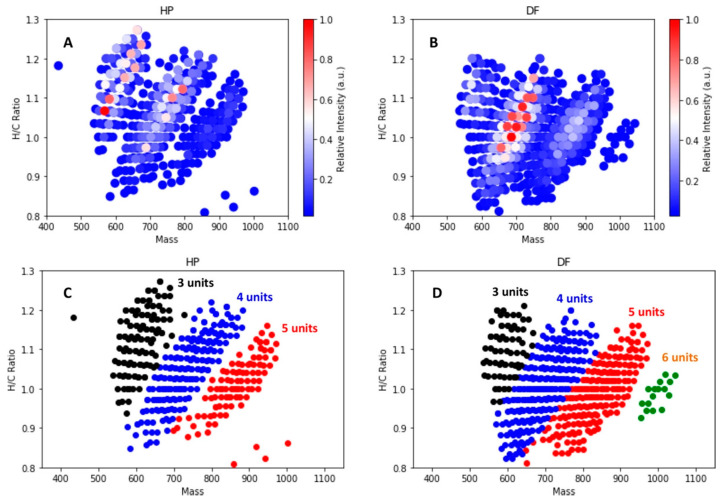
H/C ratio vs. nominal mass for the studied lignins: (**A**) for hybrid poplar lignin with color representing relative MS intensity; (**B**) for Douglas fir with color representing relative MS intensity; (**C**) for HP with color representing clusters segregated by number of aromatic units; (**D**) for DF with color representing clusters segregated by number of aromatic units. Reprinted from Terrell et al., with permission from Wiley [71].

Recently, Terrell et al. presented the application of stochastic structure generation as an alternative to assign potential structures to lignin-related oligomers with the given chemical formulae detected by FT-ICR MS [71]. Data visualization techniques such as van Krevelen diagrams, KMD, DBE, and carbon number plots were combined and revealed that some structural feature of lignin can be elucidated, for example, the aromatic units (Figure 10), the group modifications/subtractions, and structure assignments.

In summary, high-resolution mass spectrometry such as FT-ICR MS offers new possibilities for the analysis of complex mixtures, permitting the assignment of elemental formulae to detected *m/z* signals. Nevertheless, assigning a specific molecular structure to a given formula and distinguishing isomers still remains a challenge. Graphical and statistical processes eliminate some mass spectral data obfuscation and allow for a better visualization and interpretation of the lignin analyses.

## 5. Applications in Geochemistry

As an important structural component of vascular plants, lignin is chemically stable and resistant to microbial degradation, in comparison to other components such as cellulose and hemicellulose. Vascular plants are exclusively terrestrial, and this makes lignin an important contributor to soil and sedimentary organic matters. As a result, its presence in aquatic environments can serve as an unambiguous biomarker of terrigenous organic matters. In the field of geochemistry, lignin plays a significant role in tracing the biospheric carbon cycle [72,73].

**Figure 11:**
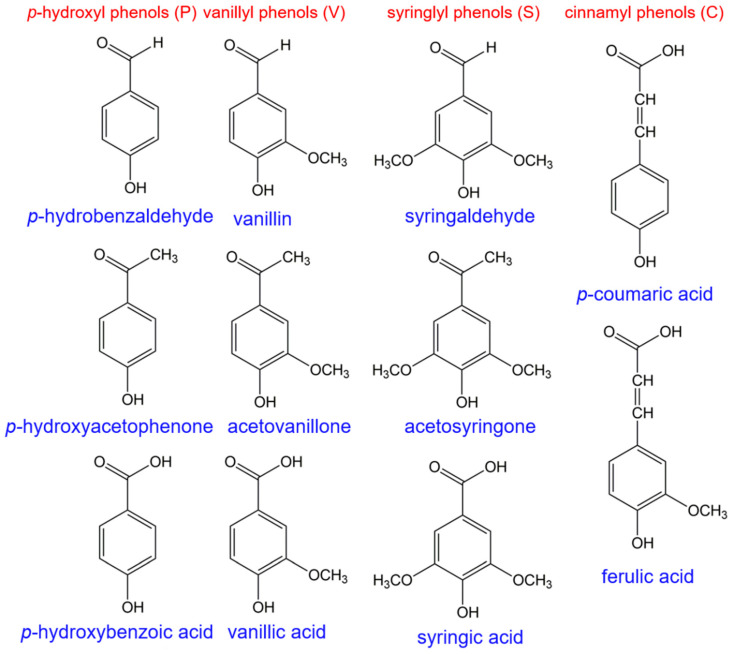
Eleven phenol monomers produced by the CuO oxidation method.

In 1982, Hedges et al. described a sensitivity and reproducible method to characterize lignin in the geochemical samples using capillary gas chromatography (GC) [74]. The entire sample was first treated with alkaline cupric oxide at 170 °C for degradation to produce simple lignin-derived monomers that were extracted with ethyl ether and then analyzed by GC on fused silica columns. A group of up to 11 phenols (Figure 11) was produced and quantified to reflect the environmental sources as well as the relative concentrations of lignin compounds present in the samples. Later, Hedges coupled GC with MS to provide more detailed and complementary identifications for the diagenetic history of vascular plant tissues in soils and sedimentary deposits [75]. Additionally, following GC-MS analysis, the yields and ratios (S/V, C/V, acid/aldehyde) of the phenol monomers have been used extensively to identify the specific composition of the vascular plant tissues. These classic methods have been modified and utilized for over 30 years to quantify terrestrially derived organic matters in environmental matrices, such as soils, sediments, and particulate/dissolved samples [76,77].

**Figure 12:**
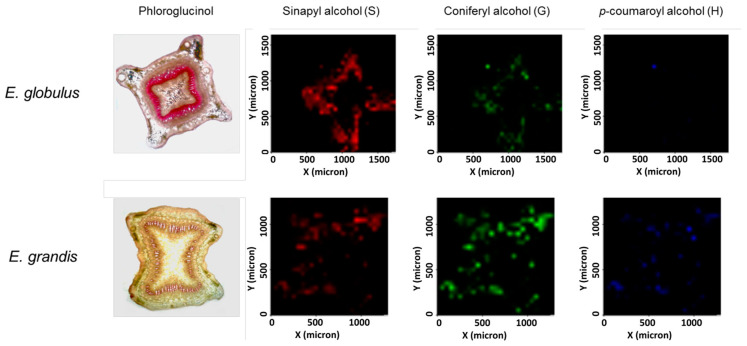
Phloroglucinol staining and mapping of sinapyl, coniferyl, and p-coumaroyl alcohols in freshly hand-cut sections of Eucalyptus stems. For relative quantification, the figures were converted to gray images. Reprinted from Mazzafera et al.,with permission from the ACS Publications [81].

Nowadays, even advanced mass spectrometry imaging (MSI) techniques have been used for mapping the spatial and lateral distributions of soluble lignins in stem, wood, and cell wall [78,79,80]. For example, hand-cut sections of stems of Eucalyptus grandis and Eucalyptus globulus were covered with silica and directly analyzed by MALDI-MSI [81]. The proportions of syringyl and guaiacyl monolignols were detected and relatively quantified (Figure 12).

## 6. Conclusions

The goal of this review was to provide the state-of-the-art of MS analysis of lignin. Mostly, API methods are used for ionization to study lignin compounds, usually in the negative ion mode. MALDI can also be applied as a shotgun method to quickly visualize the abundant compounds in various samples. Due to the complexity of the lignin composition, comprehensive ionization methods are recommended to ensure broader characterization and more accurate interpretation. In addition, HPLC combined with MS*^n^* has been widely applied for the investigation of lignin species. Coupled with CID, the real molecular weights, valuable structural information, and reaction pathways can be obtained. Due to the lack of MS libraries for lignin compounds, synthesizing artificial compounds to establish a “do-it-yourself” database or non-targeted LC-MS*^n^* strategy have been employed for the identification of lignin oligomers. To dig deeper into the mass spectral datasets, in order to reveal “hidden” information from the HRMS analyses, the concept of KMD plots has been expanded to “2D mass defect plots” by providing systematic line-ups of the different lignin linkages. Several data visualization techniques were combined, which facilitated the elucidation of structural elements of lignin species. Finally, MS of lignin has also been regarded as a powerful tool to trace the biospheric carbon cycle.

## Data Availability

The data used to support the findings of this study are available from the corresponding author upon request.
